# Understanding the neural mechanisms of empathy toward robots to shape future applications

**DOI:** 10.3389/fnbot.2023.1145989

**Published:** 2023-04-12

**Authors:** Jenna H. Chin, Kerstin S. Haring, Pilyoung Kim

**Affiliations:** ^1^Family and Child Neuroscience Lab, Department of Psychology, Brain, Artificial Intelligence, and Child (BAIC) Center, University of Denver, Denver, CO, United States; ^2^Humane Robot Technology (HuRoT) Laboratory, Department of Computer Science, Ritchie School of Engineering and Computer Science, University of Denver, Denver, CO, United States; ^3^Department of Psychology, Ewha Womans University, Seoul, Republic of Korea

**Keywords:** robotics, fNIRS, neuroscience, social robots, empathy

## Abstract

This article provides an overview on how modern neuroscience evaluations link to robot empathy. It evaluates the brain correlates of empathy and caregiving, and how they may be related to the higher functions with an emphasis on women. We discuss that the understanding of the brain correlates can inform the development of social robots with enhanced empathy and caregiving abilities. We propose that the availability of these robots will benefit many aspects of the society including transition to parenthood and parenting, in which women are deeply involved in real life and scientific research. We conclude with some of the barriers for women in the field and how robotics and robot empathy research benefits from a broad representation of researchers.

## 1. Introduction

Neurorobotics is an emergent interdisciplinary field including neuroscience, cognitive science, psychology, robotics, and artificial intelligence. For the purpose of this article, we take the following bidirectional perspective on neurorobotics: Robot capabilities can be informed by neuroscience (e.g., brain-inspired algorithms), and the human brain can be evaluated through robots. We are considering neurorobotics from the perspective of what happens in the human brain when people interact with robots. Variations in robot design, including external appearance, movement, and behavior, in addition to robot reactions to external inputs, elicit distinctive and measurable activations in the human brain. Within the context of human–robot interactions, neuroscientific approaches have been applied to developing and understanding empathy toward robots.

This is a rapidly growing field as robots become increasingly capable of advanced social skills. Consequently, robots are more prevalent in domains such as caregiving, teaching, and companionship. There is emerging literature about why and how humans empathize with robots. In this article, we review the current literature on human empathy toward robots and discuss the neural mechanisms that underlie this cognitive process. Furthermore, we propose that increased representation of female scientists is critically needed for advancements in empathetic human–robot interactions. Recent literature suggests that women have particularly sensitive neural mechanisms for empathy and caregiving, which may lead to a greater tendency to empathize with robots. Next, we discuss important considerations for researchers in the development of robot's advanced empathetic abilities. We propose that the availability of robots with advanced empathetic capabilities will assist parents in the transition to parenthood, benefit women in the society more broadly, and highlight the participation of female researchers in the field. Finally, we highlight the necessity for the use of inclusive language and the importance of representation of diverse groups that are historically underrepresented in the Neurorobotic research.

For the purpose of this study, we are focusing one particular group that is underrepresented in the Science, Technology, Engineering, and Mathematics (STEM) field, women, who make up only 28% of the STEM workforce, and even less in the robotics-related fields of Computer Occupations (25.2%) and Engineering (16.5%) (AAAU, 2020). These data refer broadly to all who self-identify as a woman. The terms *woman* and *female* are used interchangeably in this article. However, we acknowledge that 1) not all individuals who are assigned female at birth identify as women and 2) gender and sex include a broader spectrum than the terms used here.

## 2. Human empathy toward robots

Human empathy toward robots refers to the cognitive capacity of humans to perceive robots as if they have mental and emotional states, similar to those of another human. Understanding human empathy toward robots could be a crucial factor in recognizing or even predicting how people respond to robots and how robots can effectively respond to people. While this research area is a novel field, there are emerging studies that attempt to assess empathy toward robots. In the following, we review studies that sought to investigate this novel field in robotics. Because there is currently no established methodology and robots may vastly differ across research, the results of these studies may not necessarily be directly compared with each other. If methods are a concern, the exact methodology for each study should be reviewed in the cited literature.

Empathy is defined as an “other-oriented emotion elicited by and congruent with the perceived welfare of someone in need” (Batson, 2018). Empathy is a basis of emotions and behavioral responses including sympathy, empathic concerns, and compassion that are more other-oriented (Batson and Shaw, 1991; Nussbaum, 1996). In response to another's suffering, empathy is a vicarious experience of the person's feeling and empathic concerns are the concerns for the person which further motivate compassion. Compassion includes the behavioral responses to others to reduce the person's suffering (Goetz et al., 2010). In this paper, we will use empathy as the term that most broadly includes the other-oriented feelings that can lead to the motivation to help others.

Prior studies indicate that a robot showing empathetic behaviors through facial expressions and utterances was perceived differently (i.e., more friendly), supporting the idea that empathy plays a role in human–robot interactions. It seems that people empathize with robots. For example, it was stated that empathy is often seen as the basis of social cooperation and prosocial behavior and that robots capable of eliciting empathy in humans are a more successful technology (Leite et al., 2013).

How people empathize with robots depends on the human-likeness of the robot. Human-likeness in robotics describes the degree to which robots have the same features as humans [e.g., facial features like eyes, body manipulators like legs or arms, and surface looks like skin (Phillips et al., 2018)]. People empathize less with mechanical looking robots and more with human-like looking robots (Riek et al., 2009). A study examining punishment of a robot showed that not only do a robot's human-like features influence empathy but also its perceived agency (Kwak et al., 2013). Empathy toward a robot also seems to increase when the robot expresses emotions and mirrors expressions in comparison to the same robot not expressing emotions (Gonsior et al., 2012). More accurately expressed, empathy also seems to lead to robots (iCat) being perceived as more dependable and trustworthy (Cramer et al., 2010). Another study found that people had a more empathic response for a robot than a robot on a screen in a scenario where a robot first showed some intelligence and then expressed a fear (i.e., loosing its memory due to a functional problem; Seo et al., 2015).

Empathetic responses toward robot pain is a specific variety of empathy toward robots that involves recognizing a robot as if they were able to experience pain similar to how a human would. It seems that people readily perceive robots as being able to experience pain. Using fMRI, it has been shown that when observing another human in pain, the human brain processes empathy in a similar way than it processes pain (we discuss this in more detail in the next section).

### 2.1. How empathy for robots affect human behaviors

Humans have the ability to empathize with robot pain (Suzuki et al., 2015) and express concern and pity for robots that are tortured (Rosenthal-von der Pütten et al., 2013). The empathetic concern for robots leads to more hesitation to strike a robot (Darling et al., 2015). It also has been shown that inducing empathy triggers prosocial human behavior (i.e., increased helpfulness) toward a robot (Kühnlenz et al., 2013), that people seem to be inclined to help a robot find its way (Weiss et al., 2010), and that they indeed empathize with a robot when something bad happens to it (Seo et al., 2015).

However, because humans empathize with robot pain, some argue that the robots do not necessarily need to show social capabilities (Hoffman et al., 2015; Mattiassi et al., 2019). This has been reflected in the language that is used by soldiers feeling for their bomb disposal robots when they got damaged (“injured”) or destroyed (“died”). Furthermore, the notion that showing robot pain elicits human empathy should not be interpreted as a blanket application of robots. For example, it has been shown that the empathetic response to pain stimuli is modulated by emotional context (Han et al., 2009). When a robot is highly human-like (i.e., a nearly human-identical android robot), it seems that the opposite effect takes place, such that the robot is perceived less empathetic than a more machine-like robot (Złotowski et al., 2016).

Incidents of bullying robots or robot abuse have also emerged. This vocabulary is used as if we indeed feel a level of empathy toward robots. Specifically, we use the same terms we use for other agents that can experience pain (e.g., people that are being bullied and animals that are abused) and not the terms we would use for objects (e.g, *it* got destroyed and the car was damaged). This observation of human behaviors and how language is used can be tied to a phenomenological perspective on empathy (Zahavi, 2014), which is a basic class of empathy that indicates an understanding of the other, human or robot, as capable of experience (Quick, 2022). Even if robots do not truly experience the same sensations as humans or animals (e.g., pain), our language does describe what we perceive to be happening (Coeckelbergh, 2018). An early example is the “death” of hitchBOT, a hitchhiking robot that made its way across several countries for over a year, before it was subsequently destroyed by unknown assailants (Smith and Zeller, 2017). The demise of hitchBOT elicited a public reaction (e.g., social media like Twitter) and an analysis of the reactions showed a significant negative emotional reaction to its destruction, suggesting that people had formed an emotional connection with hitchBOT and perceived its destruction as morally wrong (Fraser et al., 2019).

Further research has shown that children sometimes abuse social robots in instances where they insulted the robot, obstructed its path, and even punched or kicked the robot (Nomura et al., 2015). In the same year this study was published, an intoxicated man was arrested in Japan for kicking a Pepper robot in a store (KYODO, 2015). A study on children aged 5–9 years showed that children seemed to abuse the robot, curious as to how the robot would react or whether it would enjoy it (Nomura et al., 2015). In addition, more than half of the children stated that the robot is able to perceive their abuse. It seems that children in this age group exhibit some empathy for the robot and ascribe it the ability to experience (their abuse), but choose to engage in their behaviors anyways. When comparing the bullying of humans and robots, it was found that both instances are connoted with being wrong and immoral; however, different cognitive mechanisms for moral disengagement are used (Sanoubari et al., 2021). The study also showed that, while perceived as wrong, robot mistreatment was associated with bullying less strongly and that people are less likely to intervene when a robot is being bullied.

This does raise research questions on how similarly people feel empathy for robots when compared to humans, what the differences are, and what potential significant factors could decrease or increase empathy for a robot. It has been suggested that empathy as a psychological phenomenon rooted in biology is both agile and fragile and can be broken by social change and enhanced and redirected by novel experiences (Heyes, 2018). It seems to be possible that even adults can re-learn and unlearn to empathize more or less intensively, and it could be the case that more ubiquitous robots, increased robot exposures, and new robot designs shape empathy toward robots.

Here, we propose that neuroscientific measures for empathy toward robots could lead to significantly new insights on the field of human–robot interaction as well as the field of neuroscience as robots are a novel entity in human's lives and with that have little preconceptions.

## 3. Neural mechanisms of human empathy toward robots

### 3.1. Empathy and caregiving motivation in the human brain

Activations in the brain regions that are involved in empathy support humans' empathetic thoughts and behaviors toward robots. In comparison to other species, the literature suggests that humans demonstrate an advanced capacity for empathy. This is partly due to the greater maturation of brain regions implicated in these higher-order cognitive and emotional processes. There have been several philosophical and psychological perspectives of empathy that are closely related to the neuroscientific approach. Goldman (2009) proposed the concept of mindreading as an extended form of empathy. In mindreading, there are two levels (Goldman, 2009). Low-level mindreading is based on a mirroring process whereas high-level mindreading is based on enactment imagination, perspective-taking, and mentalizing. Neuroscientific studies have identified brain regions that support a mirroring process, called the mirror neuron system. The brain regions of the mirror neuron system are the inferior frontal gyrus, the primary motor cortex, the superior temporal sulcus, and the inferior parietal lobule (Iacoboni and Dapretto, 2006). The connected activations in this system are critically involved in mirroring or imitating another person's action. The high-level mindreading such as perspective taking, enactment imagination, or mentalizing is further supported by the brain system called the theory of mind. The brain regions of the theory of mind include the medial prefrontal cortex, posterior cingulate cortex, temporal pole, superior temporal gyrus, and temporoparietal junction. This view of two levels of mindreading is also similar to Stueber (2012)'s basic and reenactive empathy theory. Stuber argues that basic empathy is a broader conception of mirror neurons while reenactive empathy is the process of how one understands the other's action by putting oneself in the other person's shoes, and trying to reenact the other person's thoughts in one's mind (Stueber, 2012).

These views are important in understanding particularly the cognitive aspect of empathy. In addition, emotional aspects of empathy have been discussed from different views including emotional contagion and sympathy. Emotional contagions refer to automatic responses to feel and mimic other person's facial expressions or actions. Hume's and Smith's views on sympathy argue that one can feel another person's feelings without being mentally engaged in imagination or reenactment (Sayre-McCord, 2013).

Another view of empathy proposes neurobiological mechanisms of these two sub-components of human empathy—emotional empathy and cognitive empathy. Emotional empathy refers to the ability to detect others' emotions and share feelings that others feel. Emotional empathy regions primarily include brain regions that are involved in emotional information processing such as the inferior frontal gyrus, anterior insula, and anterior cingular gyrus. Cognitive empathy refers to the ability to understand what others feel and think by taking the perspective of others (Decety and Jackson, 2004). Cognitive empathy regions include brain regions that are referred to earlier as being involved in theory of mind (Shamay-Tsoory et al., 2009; Yu and Chou, 2018). The human brain is also particularly sensitive to others' experiences of pain. In one study examining this sensitivity, participants received a shock or their romantic partner received a shock. Here, both conditions similarly activated the anterior insula and the anterior cingulate cortex (Singer et al., 2004).

Humans seemingly engage some of these same neural circuitries when experiencing empathy in interactions with non-human agents. How people empathize with robots can be measured by their behaviors toward robots (Spatola and Wudarczyk, 2020), through explicit measures like surveys (Nomura et al., 2006; Carpinella et al., 2017), and more recently through neuroscientific measures (e.g., EEG, MRI, and fNIRS). The latter measures are believed to be less subject to biases than explicit measures (Ogunyale et al., 2018; Spatola et al., 2021) and allow for more temporal measures as they can be administered during the stimuli which is of interest to robotics research and could allow to identify the effect of robot behaviors better as opposed to measures administered after a stimuli. We are also currently conducting a neuroimaging studying using fNIRS (Pittman et al., 2022) in conjunction with robot facial expressions in embodied robots to gain new insight into the empathy and theory of mind humans form of robots and combine them with current, explicit measures.

Indeed, research utilizing multi-modal neuroimaging has begun to identify the neural signatures of empathy and broader sociocogntive processes during human interactions with robots. In an early neurocognitive examination of human–robot interactions utilizing neuroimaging methodology, researchers found early evidence of prefrontal and superior frontal cortex activations (i.e., regions implicated in theory of mind tasks) during an interpersonal behavior paradigm performed with both human and robot opponents (Hegel et al., 2008). There is also a particular interest in examining human empathy toward robot pain. In one study utilizing fMRI, researchers found similar neural activation patterns in the amygdala, insula, and inferior frontal gyrus in participants while viewing videos of both human and robot scenarios, further supporting the idea that humans employ empathy and theory of mind circuitry in response to robot pain (Rosenthal-von der Pütten et al., 2013). EEG has also been used to assess temporal aspects of neural responses to human and robot pain scenarios. In a study conducted by Suzuki et al., researchers found similar neural responses in the P3 component in both the robot and human conditions, suggesting that humans use similar top-down processing to empathize with robots (Suzuki et al., 2015). More recent studies of human–robot interactions aim to explore empathetic relationships between humans and robots beyond laboratory settings, though early findings remain mixed (Henschel et al., 2020).

While the evidence on the development of close bonds with robots is currently limited, robots with more advanced and human-like social skills may activate the brain circuits for forming intimate relationships in humans. This is due to humans having highly developed neural circuitry related to caregiving and affiliative behaviors. The literature suggests that human caregiving involves several brain networks, primarily those involved with reward and motivation and social and emotional information processing (Kim et al., 2016; Feldman, 2017). First, caregiving relies on the perception of a wide range infant stimuli including cries, emotional faces, and gestures. Responding to these infant cues, humans form a model motivational network, recruiting dopamine and oxytocin pathways. These regions include the ventral tegmental area, substantia nigra, the striatum, and the medial prefrontal cortex. When responding to these infant cues, caregivers must also effectively regulate their own emotions, particularly when the cues may be distressing (e.g., infant cries). Key regions involved in this process include the anterior cingulate cortex and prefrontal cortex. The amygdala, which also contains an abundance of oxytocin receptors, interacts both the reward circuitry as well as emotion regulation circuitry to further modulate motivated responses and emotional salience to infant cues. Activation in these regions are closely related to the brain circuits of empathy, and are also important in supporting affiliative behaviors in close human–human relationships beyond parenting, including romantic relationships and friendships (Eslinger et al., 2021).

### 3.2. Sex differences in empathy and caregiving motivation

Individual differences may further influence how humans perceive, interact, or even develop close bonds with robots. For instance, research has found evidence for biological sex differences in social cognitive processes. Notably, differences in emotional sensitivity have been observed, such that women are more sensitive to fearful and sad stimuli whereas men are more sensitive to anger-provoking stimuli (He et al., 2018; Li et al., 2020). Previous work has also found differences in emotion identification between men and women. One meta-analysis found that while women may demonstrate a slight advantage in emotion recognition, this effect is moderated by factors such as the specific emotion and whether the emotion is positive or negative (Thompson and Voyer, 2014).

Prior research has shown that women, in comparison to men, demonstrate a greater neural response to social information (e.g., faces and people) opposed to inanimate stimuli (Proverbio et al., 2008). Relatedly, studies have indicated that women are more likely to see faces in objects (Zhou and Meng, 2020). Together, these enhancements in broad social domains set the stage for differences in later lower order processes. For instance, there may be functional differences in empathy as well as theory of mind associated with biological sex. Behavioral measures of empathy also suggest that women score higher on self-report questionnaire measures of empathy (Eisenberg and Lennon, 1983). There is also EEG evidence showing differential neural responses to pain between male and female participants such that women are more reactive to vicarious experiences of pain (Han et al., 2008). In addition, other studies have demonstrated differences in the sub-components of these processes. For instance, one study showed that women performed better than men on an affective Theory of Mind task (Baron-Cohen et al., 2003). Overall, prior research demonstrates that women may have differential brain activations and enhanced performance in cognitive domains that underlie human–robot interactions.

Even more, women may also have greater affiliative bonds with robots due to biological sex differences that underlie caregiving behaviors. Indeed, caregiving is a primary domain in which prior literature suggests differences between male and female behaviors. Evolutionary perspectives of psychobiology suggest that these differences are crucial to reproductive processes such as in sexual mate selection and subsequent caregiving. Furthermore, biophysiological changes during pregnancy also increase positive caregiving behaviors in female animals and human birthing parents. One such evolutionarily advantageous adaptive behavior increased responses to infant cues such as faces and cries. Cognitive neuroscience literature has shown that infant faces are more perceptually salient for women than men (Hahn et al., 2013), providing evidence for the "Baby Schema Effect" in which women show enhanced affective valence and orienting toward infant or child faces in comparison to adult faces (Brosch et al., 2007). Taken together, these findings suggest that those assigned female gender at birth may be more likely to engage with robots in a more prosocial manner and further develop emotional connections with robots due to being more attuned to robot social cues and enhanced sensitivity to affiliative motivation.

In discussing these findings, it is also important to note that interactions between biological sex and social cognition are complex, and interpretations of sex-based differences in social cognition require careful consideration. Specifically, sex (e.g., female and male) is determined by biological, anatomical, and chromosomal sex characteristics whereas gender (e.g., woman, man, etc.) refers to the socially constructed roles that are associated with masculinity and femininity. Previous research, such as the studies discussed in this section, oftentimes combine definitions of biological sex and gender into a single binary categorization when describing participants. Researchers in future studies should extend upon these findings by utilizing a more precise and inclusive approach to understanding the interplay among, biological sex, social constructions of gender, and social cognitive functioning.

## 4. Empathetic abilities in robots

As humans have empathy toward robots, humans respond positively to robots with empathetic abilities. In recent years, robots are providing caregiving roles in many areas including elderly care, child education, and mental health support (Broadbent, 2017; Belpaeme et al., 2018). As a robot's role in caregiving grows, evidence suggests that the empathetic abilities of the robots increase their effectiveness in caregiving roles for humans. For example, their empathetic abilities lead to more positive relationships between humans and robots (Broadbent, 2017). It was also shown that children's learning was more effectively improved when interacting with robots that are more empathetic than not empathetic (Leite et al., 2014; Marchetti et al., 2018; Kory-Westlund and Breazeal, 2019).

As discussed earlier, empathy in humans is defined by two primary components—emotional empathy and cognitive empathy. Both components—emotional empathy and cognitive empathy—are critical for appropriate and natural empathetic responses in human social interactions. Therefore, a robot's empathetic abilities in interactions with humans may require both emotional empathy and cognitive empathy. Previous studies suggest that for a robot's empathetic capacity, a robot needs to (1) model a human's emotion and (2) adjust its social behaviors based on the human's emotion (Leite et al., 2014). The first aspect is closely aligned with emotional empathy while the second aspect requires cognitive empathy. There have been studies that robots mirrored humans' emotions or provide empathetic responses to humans in specific contexts such as children playing games together with a robot (Leite et al., 2013). For more complex interactions with a wide range of contexts and individuals, robots not only need to mirror humans' emotions but also should be able to provide cognitive empathy by taking humans' internal perspectives (Asada, 2015). This is a challenging task for robots because, for effective and accurate perspective-taking, a robot needs to understand the background information of the human such as demographic factors, personality, previous experiences, and the context of the situation. Therefore, more work in this area is needed. Mirroring how human empathy is processed and generated in the brain, it will be critical for robots to be able to integrate the two components of emotional empathy and cognitive empathy.

### 4.1. An area that robots with empathetic abilities benefit women

The advanced robot's empathetic abilities are important in caregiving roles in a wide range of areas. We would like to propose one area where empathetic robots could significantly contribute; however, this area is currently receiving less attention than other applications: The robot's potential role is to support those who are in the transition to becoming new parents. This is also the area where women would particularly benefit more from the availability of the robot and the role of female scientists may also be important in developing robots with empathetic abilities. We used the gendered terms “mothers” and “fathers” in this section to match the findings of previous studies; however, we acknowledge that not all birthing parents identify themselves as “mothers” and “women.”

Having a new baby is an exciting event for new mothers, but it is also an event that often causes high levels of stress to mothers. Due to the financial, physical, and emotional demands of caring for young infants, many mothers experience a significant decrease in their wellbeing (Kim, 2021). For example, nearly 20% of new mothers experience depression or anxiety during the perinatal period (Gavin et al., 2005; Sidebottom et al., 2021). A leading cause of postpartum death is drug-related death and suicide and their risks are associated with the postpartum psychopathology (Goldman-Mellor and Margerison, 2019). What is even more concerning is that postpartum depression is one of the most untreated disorders (Marcus and Heringhausen, 2009; Vigod et al., 2016). Postpartum psychopathology is concerning not only because of the mother's own wellbeing but also because of its negative impacts on the infant's brain, cognitive, and socioemotional development (Brand and Brennan, 2009; Kingston et al., 2012; Monk et al., 2012; Waters et al., 2014; Goodman, 2019).

Mothers are often isolated due to the constant demands of caring for new infants and also feel guilt in expressing negative thoughts and feelings about being a parent. These risk factors are associated with why stress that mothers have can lead to more severe psychopathology and their conditions are undertreated. Here, robots with advanced empathetic abilities can help to detect the symptoms of postpartum psychopathology and provide the support that mothers need to cope with their stress. Robots can detect symptoms of depression and suicidal thoughts and connect those mothers to the appropriate healthcare providers. With the accurate detection of the symptoms, robots can also provide emotional support that the mothers need to reduce their stress during this stressful time.

Indeed, in the psychology and psychiatry literature, social support from a partner, family members, or friends is one of the best protective factors for preventing and reducing the symptoms of postpartum depression (Sherbourne and Stewart, 1991). Social support includes two different types—one is emotional support and the other is instrumental support. Robots that have advanced empathetic abilities can contribute to providing both types of support. For emotional support, robots can detect the mothers' moods and thoughts and provide empathetic responses to the mothers. The robots can also provide suggestions on adaptive coping strategies and share stories of other mothers who share similar experiences. Instrumental supports are a type of support that can help to care for infants. Robots with empathetic caregiving abilities can help new mothers to navigate challenges in caring for infants, for example, help understand the meaning of baby cry sounds and make suggestions on how to care for infants.

The role of support for the robot will benefit both mothers and fathers. However, it is still in the US, mothers spend significantly more hours caring for children compared to fathers (Aguiar and Hurst, 2007; Zamarro and Prados, 2021). Also, women are more vulnerable to postpartum psychopathology compared to men (Albert, 2015; Kuehner, 2017). Therefore, the availability of a robot is critically needed to support mothers, and women researchers may provide a deeper understanding of what is needed for developing robots with empathetic abilities.

## 5. Discussion and suggestions for future directions

In this perspective study, we reviewed the neural mechanisms of human empathy toward robots and the potentials for robots with empathetic abilities in assisting humans. We proposed that women who have more sensitive neural systems for empathy and caregiving compared to men are likely to exhibit the higher levels of empathy toward robots. We also proposed that robots with greater empathetic abilities may assist women, including their transition to parenthood. In studying both human empathy toward robots and robots' empathetic abilities, more inclusion of female researchers will be critical.

Here, we also propose additional considerations for future studies when discussing the importance of the role of women in neurorobotic research. First, biological sex and socially constructed gender categories are distinct. This understanding of the differences between biological sex and gender prompts additional consideration when interpreting prior findings on sex differences. Even more, biological sex is complex, and binary categorizations of assigned sex at birth do not capture all variations in hormonal, anatomical, and chromosomal differences (e.g., intersex individuals). Relatedly, inclusion efforts within the field of neurorobotics should not be limited to cisgender women. Inclusion and diversity within scientific research require an intersectional lens, taking into account how multiple minoritized identities may result in multiple forms of oppression and exclusion. Future research should aim to elevate the perspectives and work of women and other minoritized gender identities. Ultimately, this literature highlights the necessity for diverse perspectives in the creation of novel technologies that advance neurorobotics.

Neurorobotic research benefits from diverse representations of the researchers. It is shown very quickly that the field of robotics, as well as the field of neurorobotics suffers from under-representation. For example, looking only at women in the field, it shows that only 19% of computer science degrees in 2016 was awarded to women, in the History of Neuroscience in Autobiography and only 12% of the essays are authored by distinguished women neuroscientists, and between 1901 and 2019, the Nobel Prizes were awarded to a mere 21 women (counting Marie Curie two times) out of 615 scientists and many contributions of women have been simply left out or prize went to their male colleagues (Metitieri and Mele, 2022). When considering other underrepresented and minoritized groups in the field, including but not limited to Black, Indigenous, and People of Color (BIPOC), LGBTQ+ individuals, first-generation college graduates, immigrants, and people who identify in more than one or across these categories, the numbers look even more dire (MORAN, 2017). The way robots are created, including the robot technology and the interdisciplinary field surrounding robotics, is not appropriately balanced. As a result, robotics as a field is missing out on the opportunities that come with a broad participation in the research and development, and robot design process. It has been shown that full integration of minoritized scientists, under conditions of equitable and integrated work environments, leads to creativity, innovation, productivity, and positive reputational effects (Smith-Doerr et al., 2017). Gender gaps in STEM pursuits have been in part attributed to the STEM fields being deterring to communally oriented individuals (i.e., women and underrepresented minorities) as the fields impede goals of directly benefitting others, altruism, or collaboration (Diekman et al., 2015). A field like neurorobotics, however, can be highly communally oriented (e.g., empowering or helping others through neurorobotics) in addition to being highly interdisciplinary.

Overall, we argue that bidirectional empathy (i.e., human empathy toward robots and robot empathetic responses toward humans) is a primary factor in robot–human interactions. Prior examinations of the neural correlates of specific processes such as empathy, as well as social cognitive processes and behaviors more broadly, suggest that women and men may interact differently with robots due to differences in neural processes that underlie robot perception. To extend upon these findings, we propose caregiving to be a domain where a focus on bidirectional empathy between humans and robots to be particularly beneficial. Importantly, the design, implementation, and evaluation of such robots require increased diversity and inclusion within the field.

## Data availability statement

The original contributions presented in the study are included in the article/supplementary material, further inquiries can be directed to the corresponding author.

## Author contributions

KH and PK contributed to the conception of this article. JC wrote the first draft. JC, KH, and PK wrote sections of the manuscript. All authors contributed to manuscript revision, read, and approved the submitted version.
